# Curcumin enhances GSDME-mediated pyroptosis to potentiate PD-1/PD-L1 immune checkpoint blockade in colorectal cancer

**DOI:** 10.3389/fphar.2026.1734653

**Published:** 2026-02-02

**Authors:** Dongsheng Tan, Gengdong Li, Xiaoda Li, Weiwei Zhai, Lijia Jing

**Affiliations:** 1 College of Life Science, Northeast Forestry University, Harbin, China; 2 Jiangsu Food and Pharmaceutical Science College, Huaian, China; 3 Peking University Health Science Center, Beijing, China; 4 Jiangsu Engineering Research Center for Precision Health Medicated Food Product Development and Processing Technology, Huaian, China

**Keywords:** colorectal cancer, curcumin, GSDME, immune checkpointtherapy, pyroptosis

## Abstract

Colorectal cancer (CRC) patients with a microsatellite-stable (MSS) status exhibit poor responsiveness to PD-1/PD-L1 blockade. Pyroptosis induction may resensitize MSS tumors to PD-1/PD-L1 blockade; however, the expression of GSDME, a key executor of pyroptosis, is often downregulated in CRC. Here, curcumin (CUR), a natural polyphenol, was identified as a potentiator of GSDME-dependent pyroptosis in CRC. We discovered that CUR upregulates GSDME expression by inhibiting the ubiquitin–proteasome system (UPS) in the MSS-type CT26 and HT29 cell lines and activating the caspase-3/GSDME signalling axis, resulting in increased pyroptosis. In CT26 tumors, CUR-enhanced pyroptosis reshaped tumor-infiltrating immune subsets and potentiated the efficacy of anti–PD-1 therapy. Notably, the synergistic antitumor activity of CUR combined with PD-1 blockade in CT26 tumors is strictly dependent on the caspase-3/GSDME axis, as the therapeutic benefit was abolished in GSDME-knockout tumors. These findings establish CUR as a safe and effective adjuvant for PD-1/PD-L1 blockade in MSS CRC, particularly in tumors with low GSDME expression.

## 1 Introduction

Colorectal cancer (CRC) is one of the most common gastrointestinal malignancies, accounting for approximately 10% of newly diagnosed cancers worldwide. Despite advances in conventional treatments such as surgery, chemotherapy, and radiotherapy, patients with advanced CRC often experience poor survival and frequent disease recurrence (Morris et al., 2023; Fakih et al., 2023). This persistent clinical challenge highlights the urgent need for novel strategies to improve outcomes in patients with CRC.

PD-1/PD-L1 blockade has emerged as a pivotal cancer immunotherapy owing to its durable antitumor efficacy (Rui et al., 2023; Feng et al., 2017; Wu et al., 2022). PD-1 is expressed on activated T cells and modulates immune surveillance and antitumor activity (Iwai et al., 2017). PD-L1, expressed on tumor cells, binds to PD-1 to suppress T-cell proliferation and cytotoxicity, thereby facilitating tumor progression (Wu et al., 2019). Blockade of the PD-1/PD-L1 interaction restores T-cell function and augments antitumor immunity, representing a key mechanism underlying PD-1/PD-L1-targeted immunotherapy (Wang et al., 2019; Pang et al., 2023; Huang et al., 2022). Clinically, PD-1/PD-L1 inhibitors have demonstrated the ability to overcome targeted-therapy resistance across malignancies, with both combined and sequential strategies yielding significant gains in progression-free survival. (Sensi et al., 2024).

The efficacy of immune checkpoint blockade is constrained by numerous factors, including tumor-intrinsic features and the immunologic state of the tumor microenvironment. To overcome these clinical limitations, alleviate immunosuppression, and expand the therapeutic window, multidimensional strategies are being actively explored. These include modulation of the microbiota (Xia et al., 2025), pharmacologic or genetic downregulation of PD-L1 (Bao Y. et al., 2024; Liang et al., 2024), induction of immunogenic cell death, and the use of nanoplatforms to enhance tumor sensitivity to immunotherapy (Xie et al., 2024; Wang X. et al., 2025; Xu et al., 2025; Xu et al., 2024). Collectively, these approaches offer promising avenues for broadening the applicability and improving the overall effectiveness of immune checkpoint–based treatments.

In recent years, PD-1/PD-L1 blockade has filled critical gaps in advanced CRC treatment; however, most patients exhibit limited responses (Zhao et al., 2019; Yaghoubi et al., 2019). In clinical settings, microsatellite instability (MSI) occurs in only a small subset of CRC patients, who generally show a favourable response to PD-1/PD-L1 blockade therapy (Ganesh et al., 2019; Weng et al., 2022) Most CRC patients harbor microsatellite-stable (MSS) tumors, which are characterized by a low tumor mutational burden (TMB) and limited neoantigen formation (Bai et al., 2022). In MSS CRC, low immunogenicity fosters an immune-resistant tumor microenvironment (TME) enriched with immunosuppressive cells, thereby impairing antitumor immunity and reducing the efficacy of PD-1/PD-L1 blockade (Llosa et al., 2015).

Pyroptosis is an inflammatory form of programmed cell death mediated by the gasdermin (GSDM) family (Gao et al., 2022). Induction of tumor cell pyroptosis can reprogram the immune-resistant TME into an immune-active state by triggering intratumoral inflammation, thereby potentially overcoming resistance to PD-1/PD-L1 blockade (Gao et al., 2022; Wang et al., 2020; Bao X. et al., 2024). Remarkably, this phenomenon has been observed even in tumors with low TMB (Li et al., 2023). Gasdermin E (GSDME), a membrane pore-forming protein, is specifically activated by granzyme B and caspase-3 to induce pyroptosis (Wang et al., 2017; Liu et al., 2020; Cheng et al., 2024). A previous study revealed that tumor GSDME acts as a tumor suppressor by activating pyroptosis, enhancing antitumor immunity (Zhang et al., 2020). Nevertheless, a subset of CRC tumors exhibit low GSDME expression, which may lead to insufficient pyroptosis to activate antitumor immune responses (Tan et al., 2022).

Curcumin (CUR), a natural polyphenolic compound derived from the rhizome of *Curcuma longa* (De Waure et al., 2023). CUR exhibits anticancer activities, including inhibition of tumor proliferation, induction of cell death, suppression of angiogenesis, and prevention of metastasis (Kunnumakkara et al., 2008; Wang J. et al., 2025). Studies have demonstrated that CUR exerts cytotoxic effects on CRC cells by triggering caspase-3–mediated cell death (Cao et al., 2013; Hosseini et al., 2022; Howells et al., 2011; Shih et al., 2023). However, the role of CUR in modulating immune responses within CRC tumors through caspase-3 activation remains poorly understood.

In this study, we demonstrated that CUR increases GSDME levels in the MSS-phenotype CRC cell lines CT26 and HT29 by inhibiting the ubiquitin-proteasome system (UPS) while concurrently activating the caspase-3/GSDME axis to induce pyroptosis. CUR-enhanced pyroptosis remodelled tumor-infiltrating immune cell populations in CT26 tumors, thereby enhancing the efficacy of anti-PD-1 therapy. Most importantly, we demonstrated that the caspase-3/GSDME axis functions as a key molecular switch through which CUR enhances PD-1/PD-L1 blockade efficacy in MSS-phenotype CT26 tumors, establishing CUR as a candidate immunomodulatory adjuvant in MSS CRC with low GSDME expression.

## 2 Materials and methods

### 2.1 Antibodies and reagents

Antibodies against GSDME (#AF4016), β-actin (#AF7018), and Caspase-3 (#AF7022) were purchased from Affinity Biosciences (Changzhou, China). Antibodies against CD45 (#756971), CD3 (#564008), CD4 (#557307), CD8 (#569870), and FoxP3 (#563101) were obtained from BD Biosciences (New Jersey, US). GSDME (#13075) and ubiquitin (#10201) antibodies from Proteintech Group (Wuhan, China) were used for immunoprecipitation (IP). An anti-PD-1 antibody for blocking PD-1/PD-L1 in mice was purchased from BioXCell (#BE0273, West Lebanon, US). Curcumin (CUR, >98% purity) and decitabine (DAC, >98% purity) were obtained from Energy-Chemica (Shanghai, China). DSPE-mPEG2000 was obtained from AVT Pharmaceutical (Shanghai, China). Detection kits for biochemical markers were purchased from Jiangsu Sinnowa Medical Technology (Zhenjiang, China).

### 2.2 Clinical samples and data

Patient specimens and data were provided by Shanghai OUTDO Biotech Co., Ltd., National Engineering Research Center for Biochip, Shanghai, China. The collection and use of human tissue samples were approved by the Ethics Committee of the National Engineering Research Center, Shanghai (Approval No. SHYJS-CP-230902).

### 2.3 Cell culture and viability assay

The CT26 and HT29 cell lines were maintained in DMEM supplemented with 10% FBS and 2 mM L-glutamine at 37 °C under 5% CO_2_. Cell viability was assessed via a Cell Counting Kit-8 (APExBIO Technology, Texas, US), and extracellular LDH release was measured via an ELISA kit (Shanghai Enzyme-linked Biotechnology, Shanghai, China). The cell morphology was evaluated via microscopic examination.

### 2.4 Western blot analysis

The samples were lysed in RIPA buffer containing protease and phosphatase inhibitors (Servicebio, Wuhan, China). The lysates were subsequently centrifuged at 13,000 rpm for 15 min at 4 °C, after which the protein concentrations were determined via a BCA assay (Yeasen, Shanghai, China). Proteins were separated by SDS–PAGE, transferred to PVDF membranes, blocked with 5% skim milk, and incubated overnight at 4 °C with primary antibodies. After incubation with HRP-conjugated secondary antibodies, protein bands were visualized via enhanced chemiluminescence (ECL) (Servicebio, Wuhan, China).

### 2.5 Immunoprecipitation (IP) assay

The cells were lysed in IP buffer and centrifuged at 14,000 × g for 15 min at 4 °C, with 10% of the supernatant retained as input. The remaining lysate was incubated overnight at 4 °C with an anti-GSDME antibody and protein A/G magnetic beads (HY-K0202, MCE, New Jersey, US). The immunocomplexes were washed three times with PBS, boiled in sample buffer for 5 min, and analysed by immunoblotting with an anti-ubiquitin antibody.

### 2.6 Proteasome activity assay *in vitro*

After being washed with PBS, the cells were lysed in proteasome lysis buffer. The lysates were clarified by centrifugation at 4 °C, and the protein concentrations were quantified via a BCA assay. Equal aliquots (40 µg) of protein were dispensed into black 96-well plates and incubated with 50 µM Suc-LLVY-AMC (MCE, New Jersey, United States) at 37 °C for 60 min. Fluorescence (Ex 380 nm/Em 460 nm) was measured via a microplate reader, and proteasome activity was determined by subtracting the blank control signals.

### 2.7 Generation of *gsdme* knockout CT26 cell lines

The *Gsdme*-knockout CT26 cell line was generated as previously described (Zhang et al., 2020). *Gsdme*-targeting gRNAs were subsequently cloned and inserted into the lentiCRISPR-v2-puro vector. The plasmids were cotransfected with pSPAX2 and pCMV-VSV-G into HEK293T cells. Viral supernatants were harvested 48 h posttransfection and used to transduce CT26 cells. After 48 h, the cells were selected with puromycin (8 μg/mL).

### 2.8 Preparation of the CUR formulation

DSPE-mPEG2000 and CUR were mixed at a 10:1 mass ratio and dissolved in anhydrous ethanol in a round-bottom flask. The solution was subjected to rotary evaporation, followed by vacuum drying for 24 h. Phosphate-buffered saline (PBS) was then added, and the mixture was sonicated for 20 min to prepare the CUR formulation.

### 2.9 Animals and experimental ethics

Male BALB/c mice (6–8 weeks old) were purchased from Jiangsu Huachuang Xinnuo Pharmaceutical Technology Co., Ltd. All the animal experiments were approved by the Institutional Animal Care and Use Committee and conducted in accordance with the NIH Guide for the Care and Use of Laboratory Animals.

### 2.10 Toxicology assessment

The mice received intravenous injections of CUR or DAC at various doses for a total of 15 administrations over 30 days. After treatment, blood and tissues (heart, liver, spleen, lungs, kidneys, thymus, and femurs) were collected. Organ coefficients were calculated, and hematological, renal, and hepatic parameters were assessed. Tissues were fixed in 4% paraformaldehyde, embedded, sectioned, and stained with hematoxylin and eosin (H&E).

### 2.11 Tumor models

For the orthotopic tumor model, the mice were fasted for 12 h prior to anaesthesia. A midline abdominal incision was made to expose the colon, and CT26 cells (1 × 10^6^ cells per mouse) were injected into the colonic mucosa. The peritoneum, abdominal muscles, and skin were sequentially sutured, and the incision was disinfected with 75% ethanol. For the subcutaneous model, CT26 cells (1 × 10^6^ cells per mouse) were injected subcutaneously into the dorsal region after disinfection with 75% ethanol.

### 2.12 Proteomic analysis

Tumor tissues and cancer cell lines were lysed via sonication in 8 M urea buffer containing protease inhibitors and EDTA, followed by centrifugation to isolate the lysates. Protein concentrations were determined via a BCA assay, after which the proteins were digested into peptides, desalted, and dried prior to DIA-based LC–MS/MS analysis. Differentially expressed proteins were subjected to Gene Ontology enrichment and pathway analysis, and expression patterns were visualized as heatmaps. All the experiments were performed by OE Biotech Co., Ltd. (Shanghai, China).

### 2.13 Flow cytometry

Single-cell suspensions were prepared from excised tumors by mechanical dissociation and enzymatic digestion, followed by filtration, red blood cell lysis, and Fc receptor blocking. The cells were stained with fluorophore-conjugated antibodies against surface and intracellular markers, with compensation set using single-stained beads. The data were acquired on a Beckman Coulter CytoFLEX cytometer and analysed via FlowJo software.

### 2.14 Statistical analysis

The data were analysed and visualized via GraphPad Prism 9.0. Statistical significance among treatment groups was determined via one-way ANOVA with Tukey’s *post hoc* test or two-tailed Student’s t-test, as appropriate. The data are presented as the means ± standard errors of the means (SEMs). *P < 0.05; **P < 0.01; ***P < 0.001; ****P < 0.0001.

## 3 Results

### 3.1 Associations of GSDME with CD8^+^ T cells and PD-L1 expression in human CRC

**FIGURE 1:**
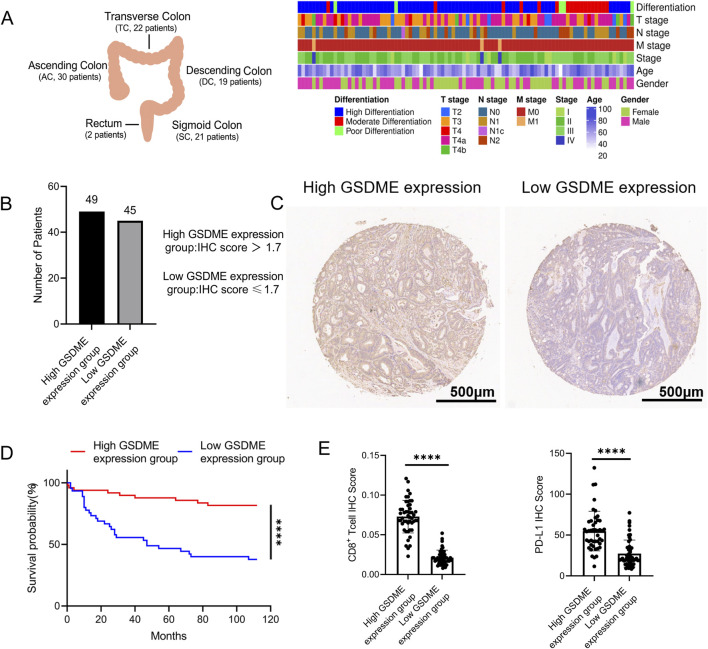
**(A)** General information of the CRC patients and their tumor specimens included in the study. **(B)** Patients were divided into high- and low-expression groups using an IHC score cutoff of 1.7. **(C)** Representative IHC images of the high- and low-expression groups. **(D)** Association between GSDME expression and overall survival of patients. **(E)** Correlation of GSDME expression with intratumoral CD8^+^ T-cell infiltration and PD-L1 expression.

In MSS CT26 tumors, GSDME promotes CD8^+^ T-cell infiltration and strengthens antitumor immune responses (Luo et al., 2024). To determine whether a similar feature exists in human MSS CRC, GSDME expression was assessed via immunohistochemistry (IHC) in tumor specimens from 94 MSS CRC patients (Figure 1A). Patients were classified into high (score >1.7, n = 49) and low (score ≤1.7, n = 45) GSDME expression groups on the basis of their IHC scores (Figures 1B,C). Survival analysis revealed markedly improved overall survival in the high-expression group, with 81.6% of patients alive at 90 months post-surgery, compared with 40.0% in the low-expression group (Figure 1D), underscoring the tumor-suppressive function of GSDME in MSS CRC. Notably, high GSDME-expressing MSS CRC tumors presented increased CD8^+^ T-cell infiltration and elevated PD-L1 expression (Figure 1E), both of which are predictive of a favourable response to PD-1/PD-L1 blockade. These findings suggest that GSDME may contribute to shaping a tumor immune landscape in human MSS CRC that favours responsiveness to PD-1/PD-L1 blockade.

### 3.2 CUR upregulates and activates GSDME to amplify pyroptosis in MSS CRC cells

**FIGURE 2:**
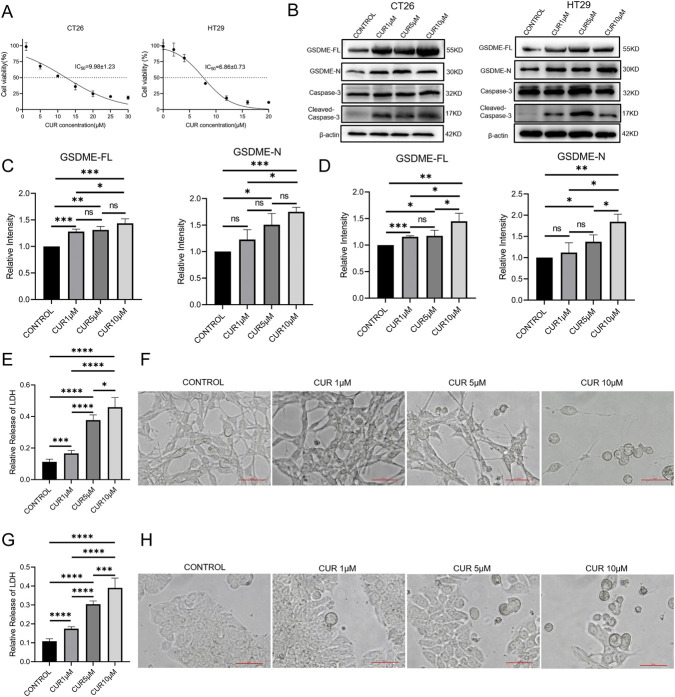
**(A)** IC_50_ values of CUR in CT26 and HT29 cells following 72 h of treatment. **(B)** Western blot analysis of full-length GSDME (GSDME-FL), GSDME-N, and cleaved caspase-3 in CT26 and HT29 cells treated with 10 μM CUR for 72 h. Quantification of GSDME-FL and GSDME-N expression in CT26 **(C)** and HT29 **(D)** cells on the basis of densitometric analysis of Western blots. LDH release in CT26 **(E)** and HT29 **(G)** cells following CUR treatment (1, 5, or 10 μM for 72 h). Microscopic visualization of pyroptotic morphology in CT26 **(F)** and HT29 **(H)** cells with or without CUR treatment (1, 5, or 10 μM for 72 h).

Mouse (CT26) and human (HT29) CRC cell lines, both of which exhibit an MSS phenotype, were treated with CUR at 1, 5, or 10 μM for 72 h, covering concentrations around the IC_50_ values for both cell lines (Figure 2A). Western blot analysis revealed the upregulation of GSDME and its pore-forming N-terminal fragment (GSDME-N) in both cell lines upon CUR treatment, with the maximal effect observed at 10 μM CUR (Figures 2B–D). Moreover, CUR treatment also activated caspase-3, which specifically cleaves GSDME to release GSDME-N (Wang et al., 2017). These results indicate that CUR upregulates GSDME and activates the caspase-3/GSDME axis to amplify pyroptosis in these two CRC cell lines.

The amplification of pyroptosis was further confirmed by assessing lactate dehydrogenase (LDH) release and cell morphology. CUR induced a concentration-dependent increase in LDH release in both cell lines (Figures 2E,G). Consistently, CUR-treated cells displayed a swollen pyroptotic morphology, with the proportion of pyroptotic cells increasing with increasing CUR concentration (Figures 2F,H). Collectively, these findings suggest that CUR enhances pyroptosis in CT26 and HT29 cells.

### 3.3 Mechanism of CUR-induced GSDME upregulation in MSS CRC cells

**FIGURE 3:**
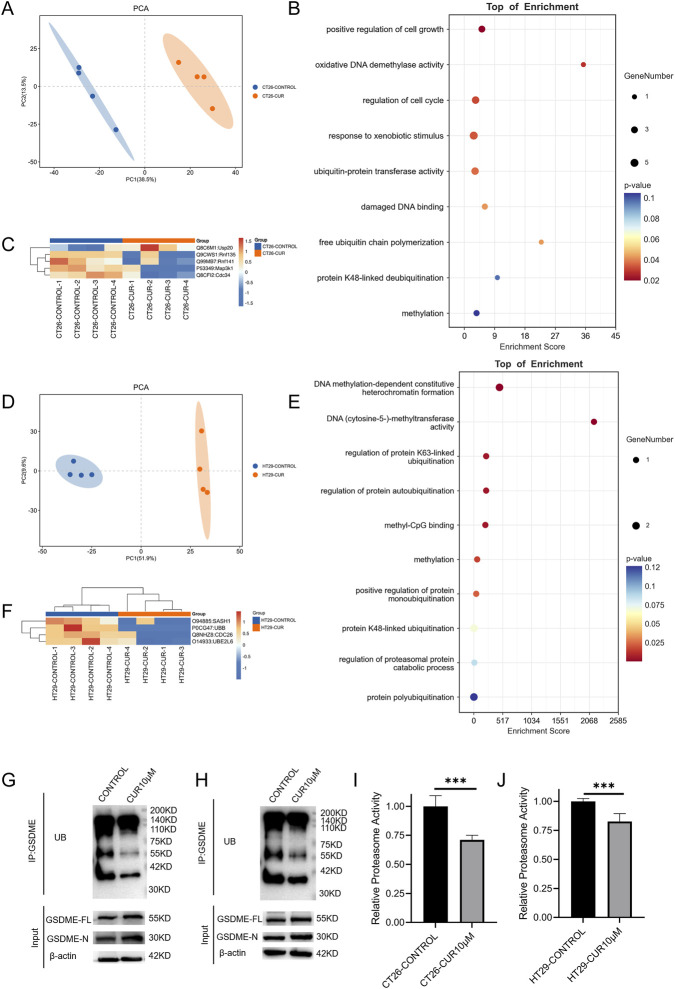
**(A)** Principal component analysis (PCA) of proteins from CT26 cells ± CUR (n = 4). GO enrichment **(B)** and cluster analysis **(C)** of DEPs in CT26 cells ± CUR. **(D)** PCA of proteins from HT29 cells ± CUR (n = 4). GO enrichment **(E)** and cluster analysis **(F)** of DEPs in HT29 cells ± CUR. **(G,H)** Immunoprecipitation (IP) analysis of GSDME ubiquitination in CT26 **(G)** and HT29 **(H)** cells treated with or without CUR (72 h, 10 μM). **(I,J)** Changes in proteasome activity in CT26 **(I)** and HT29 **(J)** cells after CUR treatment (72 h, 10 μM).

To investigate the mechanism underlying the CUR-mediated upregulation of GSDME, quantitative proteomic analysis was performed on CUR-treated CT26 and HT29 cells. Principal component analysis (PCA) revealed distinct proteomic profiles between treated and untreated cells (Figures 3A,D). Differentially expressed proteins (DEPs) were identified in each cell line using a threshold of P < 0.05 and a fold change (FC) ≥ 1.5.

GO analysis of DEPs in CT26 and HT29 cells revealed significant enrichment of intracellular processes, particularly those associated with the ubiquitin–proteasome system (UPS), which regulates intracellular protein levels (Figures 3B,E). Hierarchical clustering (Figure 3C) revealed that, following CUR treatment, Usp20 (ubiquitin-specific protease 20), a deubiquitinating enzyme (Li et al., 2022), was upregulated, whereas several ubiquitination-related enzymes, including Map3k1 (mitogen-activated protein kinase kinase kinase 1) (Suddason and Gallagher, 2015), Cdc34 (cell division cycle 34) (Jie et al., 2025), Rnf135 (RING finger protein 135) (Hu et al., 2025), and Rnf141 (RING finger protein 141) (Zhang et al., 2021), were downregulated in CT26 cells. In CUR-treated HT29 cells, SASH1 (SAM and SH3 domain-containing protein 1), UBB (ubiquitin B), CDC26 (cell division cycle 26), and UBE2L6 (ubiquitin-conjugating enzyme E2 L6) were downregulated (Figure 3F); these proteins exhibit ubiquitin ligase activity either directly or indirectly (Orfali et al., 2020; Masuda et al., 2015; Dauphinee et al., 2013; Oh et al., 2013).

Previous studies have shown that CUR broadly affects UPS activity, with specific molecular targets varying across cell types (Torghabe et al., 2025). Although proteomic data revealed distinct expression patterns of ubiquitination-related proteins in CT26 and HT29 cells following CUR treatment, these observations suggest that CUR may influence GSDME levels in both cell lines through modulation of UPS activity. Consistent with this interpretation, immunoprecipitation (IP) and proteasome activity assays revealed reduced GSDME ubiquitination and decreased proteasome activity upon CUR treatment in both cell lines (Figures 3G–J). Taken together, these findings support a working model in which CUR promotes intracellular accumulation of GSDME by attenuating its ubiquitination and subsequent proteasome-mediated turnover.

### 3.4 CUR enhances pyroptosis in CT26 tumors without apparent toxicity.

CT26 tumor-bearing mice were used to evaluate CUR-enhanced, GSDME-dependent pyroptosis *in vivo*. Decitabine (DAC), a DNA methyltransferase inhibitor known to upregulate GSDME and induce pyroptosis in various tumor types (Gong et al., 2023; Chen et al., 2025), was included as a control. The mice received daily intravenous injections of CUR or DAC at 1, 5, or 10 mg/kg for five consecutive days. To facilitate CUR administration, a hydrophilic CUR-loaded nanoformulation was prepared via DSPE-mPEG2000. This nanoformulation markedly enhanced CUR dispersion in phosphate-buffered saline and resulted in a well-defined spherical morphology, uniform particle size, and sustained CUR release (Supplementary Figures S1–S4).

**FIGURE 4:**
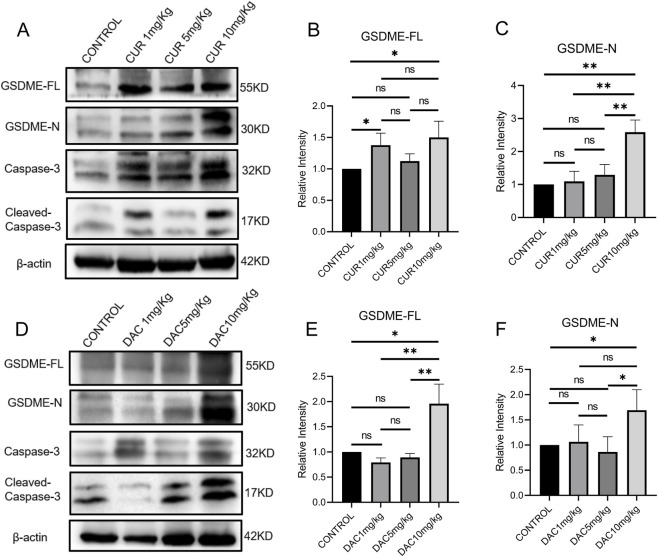
**(A)** Western blot analysis of GSDME, GSDME-N, and cleaved caspase-3 in CT26 tumors treated with CUR (10 mg/kg). Quantification of GSDME **(B)** and GSDME-N **(C)** protein levels in CUR-treated CT26 tumors. **(D)** Western blot analysis of GSDME, GSDME-N, and cleaved caspase-3 in CT26 tumors treated with DAC (10 mg/kg). Quantification of GSDME **(E)** and GSDME-N **(F)** protein levels in DAC-treated CT26 tumors.

**FIGURE 5:**
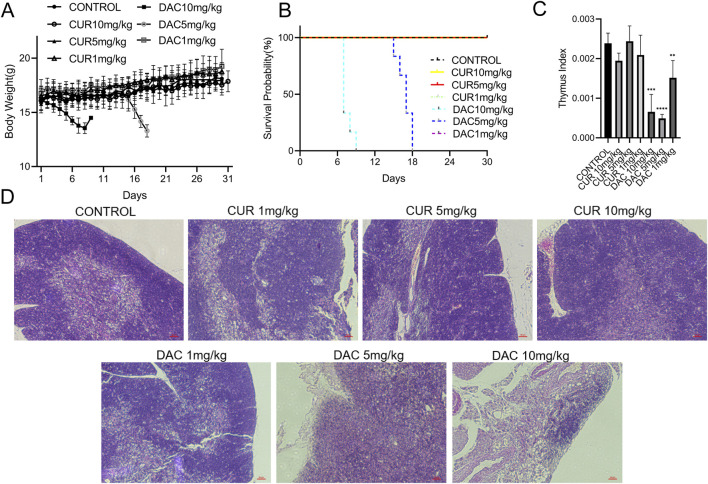
**(A)** Body weight changes in the mice in each treatment group during the study. **(B)** Survival curves of the mice in each group. **(C)** Thymus index (thymus weight-to-body weight ratio) in each group. **(D)** Representative H&E-stained sections of thymus tissue from each treatment group.

**FIGURE 6:**
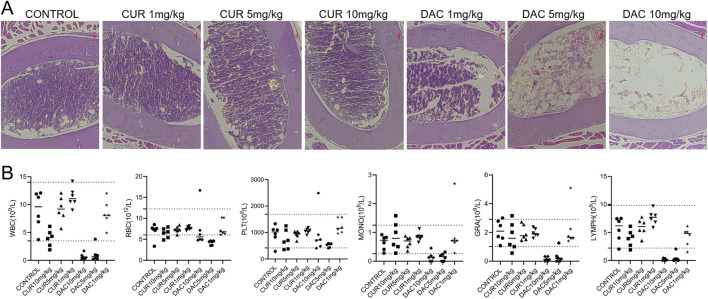
**(A)** Representative H&E-stained sections of femoral bone marrow from each treatment group. **(B)** Hematological parameters, including white blood cells (WBC), red blood cells (RBC), platelets (PLT), monocytes (MONO), granulocytes (GRA), and lymphocytes (LYMPH).

The administration of CUR or DAC at 10 mg/kg significantly upregulated and activated GSDME, respectively, in CT26 tumors (Figures 4A–F). Compared with the controls, CUR induced ∼1.5- and ∼2.6-fold increases in total GSDME and GSDME-N, respectively, whereas DAC induced ∼1.9- and ∼1.7-fold increases, comparable to those observed with CUR. To assess safety, healthy mice received intravenous CUR or DAC at 1, 5, or 10 mg/kg every other day for 30 days, with saline-treated mice serving as controls. CUR was well tolerated, as indicated by normal weight gain and the absence of mortality (Figures 5A,B). In contrast, DAC exhibited dose-dependent toxicity: treatment with 5 or 10 mg/kg resulted in significant weight loss and substantial mortality (Figures 5A,B). High-dose DAC also caused pronounced thymic atrophy, as evidenced by a reduced thymus index and loss of the medullary region enriched in immature T cells (Figures 5C,D), along with dose-dependent decreases in femoral bone marrow density and peripheral blood counts of WBCs, monocytes, granulocytes, and lymphocytes (Figures 6A,B). These results indicate that CUR effectively upregulates and activates GSDME to increase pyroptosis in CT26 tumors, with an efficacy comparable to that of DAC but minimal toxicity.

### 3.5 CUR-enhanced pyroptosis modulates the TME in CT26 tumors

**FIGURE 7:**
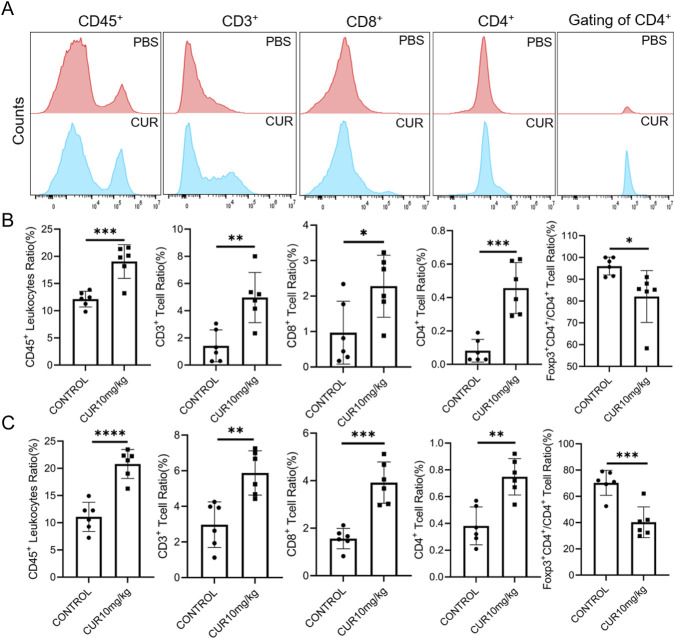
**(A)** Flow cytometric gating strategy for immune cell sorting. **(B)** Flow cytometry analysis of immune cell infiltration in subcutaneous CT26 tumors after CUR treatment. **(C)** Flow cytometry analysis of immune cell infiltration in orthotopic CT26 tumors after CUR treatment.

To assess the impact of CUR-enhanced pyroptosis on the TME in CT26 tumors, immune cell subsets were characterized by staining with fluorescently labelled antibodies against lineage-specific surface and intracellular markers (Figure 7A).

Compared with the control, CUR treatment significantly increased the proportion of CD45^+^ leukocytes among total tumor cells in both the subcutaneous and orthotopic CT26 tumor models, indicating enhanced immune cell recruitment to the tumor site (Figures 7B,C). Further analysis of T-cell subsets revealed a substantial increase in CD3^+^ T-cell infiltration following CUR treatment. Notably, the proportions of antitumor effector CD8^+^ and CD4^+^ T cells were significantly elevated, whereas the fraction of immunosuppressive regulatory T cells (Foxp3^+^CD4^+^ Tregs) within the CD4^+^ population was markedly reduced (Figures 7B,C). These findings suggest that CUR-induced pyroptosis promotes the expansion of antitumor effector cells while suppressing immunosuppressive subsets.

### 3.6 CUR potentiates PD-1/PD-L1 blockade in a CT26 tumor model

The therapeutic efficacy of CUR in combination with an anti-PD-1 monoclonal antibody was evaluated in both subcutaneous and orthotopic CT26 tumor models. CUR and anti-PD-1 were administered every 2 days for 2 weeks at 10 mg/kg each. In the subcutaneous tumor model, both agents were delivered via intravenous injection, whereas in the orthotopic model, CUR was administered orally, and anti-PD-1 was administered intraperitoneally.

**FIGURE 8:**
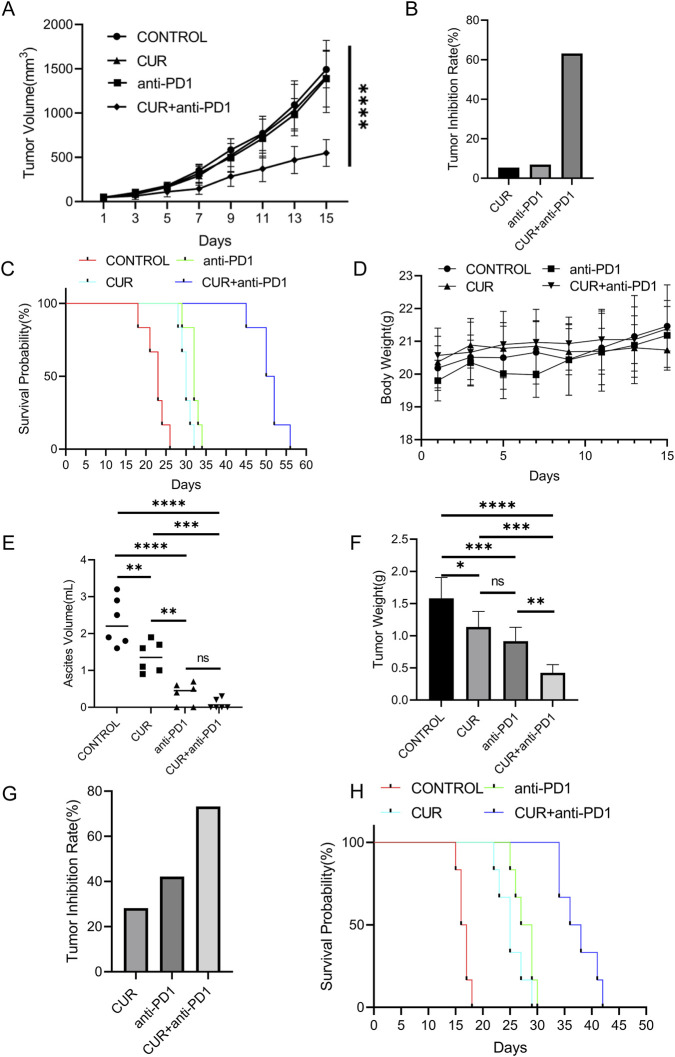
**(A)** Tumor growth curves of mice bearing subcutaneous tumors following different treatments. **(B)** Tumor inhibition rates in the subcutaneous tumor model. **(C)** Survival of mice bearing subcutaneous tumors in each treatment group. **(D)** Body weight changes in mice bearing subcutaneous tumors during treatment. **(E)** Ascite formation in mice bearing orthotopic tumors. **(F)** Orthotopic tumor weights after 15 days of treatment. **(G)** Inhibition rates of orthotopic tumors in each group. **(H)** Survival curves of mice bearing orthotopic tumors in each treatment group.

In the subcutaneous model, CUR monotherapy resulted in negligible antitumor activity, and PD-1 blockade alone was similarly ineffective because of the immunosuppressive TME (Figures 8A,B). In contrast, combination therapy with CUR and anti-PD-1 markedly suppressed tumor growth compared with either monotherapy, as reflected by reduced tumor volumes and increased tumor inhibition rates (Figures 8A,B). This combination also significantly prolonged overall survival (Figure 8C) while maintaining stable body weight throughout the treatment period (Figure 8D).

In the orthotopic model, control mice developed substantial ascites, with 50% exhibiting severe accumulation (Figure 8E). CUR monotherapy partially alleviated ascites, whereas anti-PD-1 therapy alone and combination therapy achieved greater reductions. The mean tumor weights were lowest in the combination group, intermediate in the anti-PD-1 and CUR groups, and highest in the control group (Figure 8F). The tumor inhibition rates were 28.2% (CUR), 42.2% (anti-PD-1), and 73.3% (combination) (Figure 8G), demonstrating that CUR potentiates PD-1 blockade. Moreover, the combination treatment significantly prolonged overall survival compared with either monotherapy (Figure 8H).

### 3.7 GSDME as a key mediator of CUR–PD-1 synergy in CT26 tumors

**FIGURE 9:**
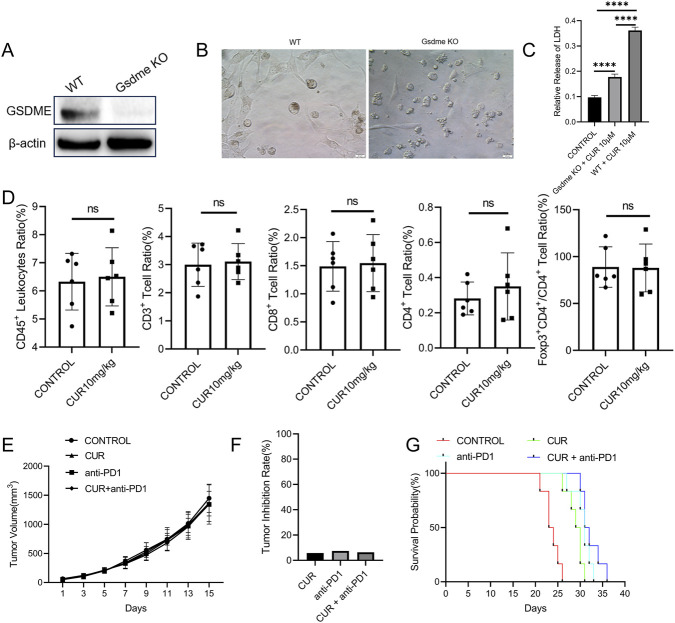
**(A)** Western blot showing GSDME expression in the Gsdme-KO CT26 cell line. **(B)** Cell morphology and **(C)** LDH release profiles of the WT and Gsdme-KO CT26 cell lines after CUR treatment (10 μM, 72 h). **(D)** Flow cytometry analysis of immune cell infiltration in subcutaneous Gsdme-KO CT26 tumors after CUR treatment. Tumor growth curves under different treatments **(E)**, tumor inhibition rates **(F)**, and survival rates of Gsdme-KO tumor–bearing mice **(G)**.

To confirm the essential role of GSDME in the synergistic effect of CUR and PD-1 blockade, subcutaneous tumors were established via the use of GSDME-knockout CT26 cells (Figure 9A). Loss of GSDME abrogated CUR-induced pyroptosis in CT26 cells, as indicated by diminished LDH release and an apoptotic morphology instead of pyroptotic features (Figures 9B,C). In GSDME-deficient tumors, CUR failed to reprogram immune cell subsets or synergize with PD-1 blockade (Figures 9D–G). These results demonstrate that the cooperative antitumor efficacy of CUR and PD-1 blockade in CT26 tumors is critically dependent on GSDME-mediated tumor cell pyroptosis.

## 4 Discussion

In this study, we demonstrated that CUR enhances antitumor immunity in MSS CRC and effectively sensitizes tumors to PD-1 blockade. Mechanistically, our findings provide two key insights: (i) CUR upregulates GSDME by suppressing the UPS, thereby activating the caspase-3/GSDME axis to amplify pyroptosis; and (ii) CUR-induced pyroptosis reshapes the tumor immune microenvironment (TME), ultimately improving responsiveness to PD-1 blockade.

GSDME is a pivotal determinant of cell death, redirecting apoptosis toward pyroptosis and thereby influencing antitumor immune activation (Hu et al., 2020; Wu et al., 2025; Zhang et al., 2019). However, GSDME is frequently silenced in CRC. In our patient cohort, high GSDME expression was associated with markedly improved overall survival and increased CD8^+^ T-cell infiltration in MSS CRC tumors. Our findings from the CT26 tumor model are consistent with a role for GSDME-dependent pyroptosis in promoting an inflamed, cytotoxic T cell–rich tumor microenvironment. Together, these data suggest that insufficient GSDME expression may represent an underrecognized contributor to the poor clinical performance of immune checkpoint inhibitors in MSS CRC.

We further showed that CUR robustly enhances GSDME expression and promotes the generation of its pore-forming N-terminal fragment in MSS-phenotype CRC cells. This induction coincides with caspase-3 activation and classical pyroptotic features, including LDH release and cellular swelling. Mechanistically, CUR reduces GSDME ubiquitination and prevents its proteasomal degradation, as supported by proteomic profiling and immunoprecipitation analyses. Considering that GSDME downregulation is prevalent in a subset of CRC tumors, these findings provide a mechanistic explanation for the restoration of pyroptotic competence in MSS CRC cells following CUR treatment.

DNA methyltransferase inhibitors such as DAC can restore GSDME transcription by reversing promoter hypermethylation and have shown therapeutic benefit across multiple cancers, including CRC (Gong et al., 2023; Zhou et al., 2024). However, the effectiveness of DAC is limited by substantial toxicity, particularly myelosuppression and immune impairment. In contrast, CUR enhances GSDME-mediated pyroptosis without eliciting hematopoietic or thymic injury. This distinction is clinically meaningful, as preserved bone-marrow and thymic function are essential for sustaining antitumor immunity and ensuring the effectiveness of T-cell–dependent immunotherapies such as PD-1/PD-L1 blockade (Cardinale et al., 2021; Velardi et al., 2021).

The CT26 MSS tumor model is intrinsically refractory to PD-1 blockade because of its suppressive effect on the TME. Notably, CUR-driven pyroptosis reprogrammed the TME by increasing CD8^+^ and CD4^+^ T-cell infiltration while depleting Tregs, thereby creating a permissive immune landscape for PD-1 therapy. Although CUR has been reported to potentiate PD-1/PD-L1 therapy through the modulation of cancer-associated fibroblasts (Hou et al., 2025), our results establish that its synergistic efficacy strictly requires the caspase-3/GSDME axis, as the therapeutic benefit was completely lost in GSDME-knockout tumors. These findings identify the caspase-3/GSDME axis as a central immunomodulatory switch through which CUR orchestrates TME remodeling and enhances responsiveness to PD-1/PD-L1 blockade. Collectively, our results highlight not only a previously unrecognized role of CUR in modulating immunity in MSS CRC but also the caspase-3/GSDME axis as a promising therapeutic target for sensitizing MSS CRC to immune checkpoint inhibition.

Despite these encouraging findings, several limitations should be acknowledged. First, although our proteomic and biochemical analyses indicate that CUR-mediated inhibition of the UPS contributes to the regulation of GSDME abundance in both CT26 and HT29 cells, the specific molecular target of CUR within the UPS has not yet been identified. Future studies will focus on pinpointing the UPS component directly targeted by CUR and clarifying the underlying regulatory mechanisms.

In addition, the enhancement of PD-1 responsiveness by CUR-induced pyroptosis has so far been demonstrated only in the murine CT26 model. To improve the translational relevance of these findings, future investigations will incorporate mouse models with humanized immune systems and humanized MSS CRC tumor models to further validate the therapeutic potential of CUR.

**FIGURE 10:**
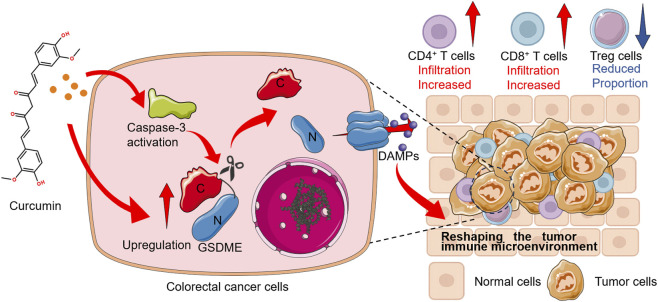
Schematic illustration of the mechanism by which CUR remodels the immunosuppressive TME in CRC by amplifying GSDME-dependent pyroptosis.

In summary, our results indicate that CUR could function as a safe immunomodulatory adjunct with the potential to improve PD-1/PD-L1 immunotherapy in MSS CRC by augmenting GSDME-dependent pyroptosis and helping to reshape the TME (Figure 10). These results provide mechanistic insight into the role of CUR in tumor immunomodulation and highlight a clinically translatable strategy for enhancing personalized PD-1/PD-L1 therapy in MSS CRC tumors with low GSDME expression.

## Data Availability

The original contributions presented in the study are included in the article/Supplementary Material, further inquiries can be directed to the corresponding authors.
