# Supplementary material for: Curcumin enhances GSDME-mediated pyroptosis to potentiate PD-1/PD-L1 immune checkpoint blockade in colorectal cancer
Source: Front Pharmacol. 2026 Feb 2;17:1734653. doi: 10.3389/fphar.2026.1734653 (PMC12907348; doi:10.3389/fphar.2026.1734653)
Supplement: Supplementary file 1 [file DataSheet1.pdf]

Supplementary materials

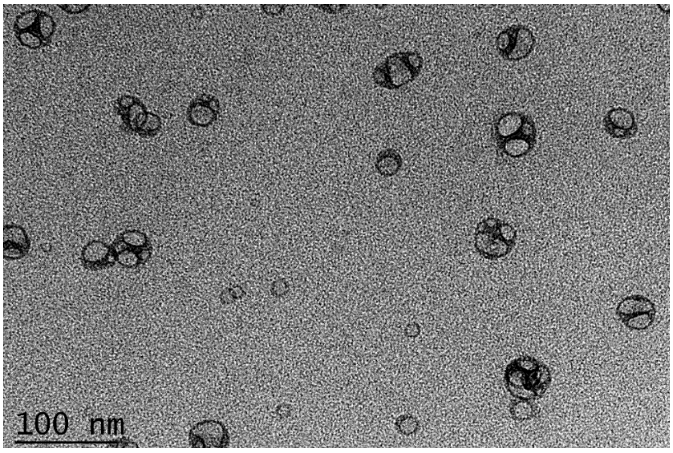

Figure S1. Representative TEM image of CUR-loaded nanformulation.

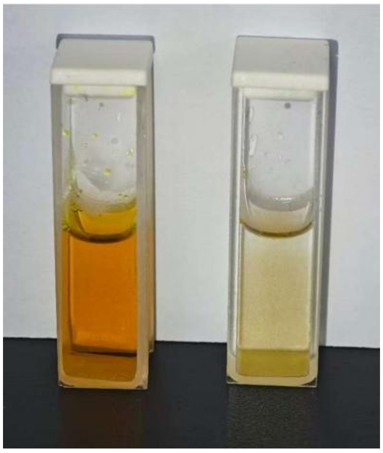

Figure S2. Visual comparison of the dispersibility between free CUR and CUR-loaded nanoformulation in PBS.

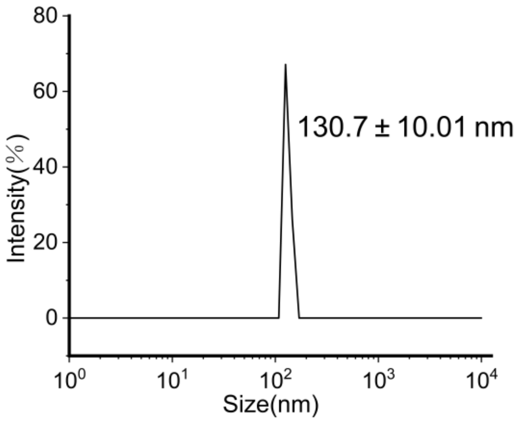

Figure S3. Hydrodynamic diameter of CUR-loaded nanoformulation measured by DLS analysis.

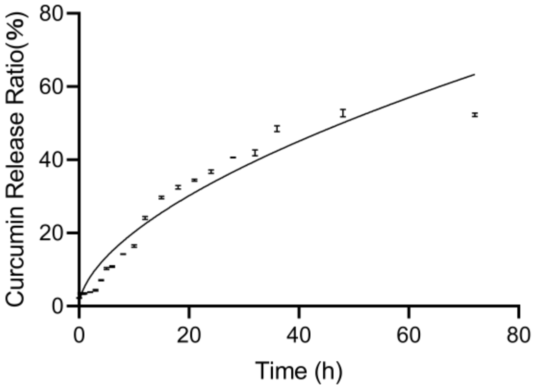

Figure S4. *In vitro* release profile of CUR from CUR-loaded nanoformulation under simulated physiological conditions (PBS, pH 7.4, 37 °C).
